# Optimization of Cat's Whiskers Tea (*Orthosiphon stamineus*) Using Supercritical Carbon Dioxide and Selective Chemotherapeutic Potential against Prostate Cancer Cells

**DOI:** 10.1155/2014/396016

**Published:** 2014-09-07

**Authors:** Fouad Saleih R. Al-Suede, Mohamed B. Khadeer Ahamed, Aman S. Abdul Majid, Hussin M. Baharetha, Loiy E. A. Hassan, Mohd Omar A. Kadir, Zeyad D. Nassar, Amin M. S. Abdul Majid

**Affiliations:** ^1^EMAN Research and Testing Laboratories, School of Pharmaceutical Sciences, Universiti Sains Malaysia, 11800 Minden, Pulau Pinang, Malaysia; ^2^Advanced Medical and Dental Institute (IPPT), Universiti Sains Malaysia, 11800 Minden, Pulau Pinang, Malaysia; ^3^School of Industrial Technology, Universiti Sains Malaysia, 11800 Minden, Pulau Pinang, Malaysia; ^4^School of Pharmacy, The University of Queensland, 20 Cornwall Street, Woolloongabba, QLD 4102, Australia

## Abstract

Cat's whiskers (*Orthosiphon stamineus*) leaves extracts were prepared using supercritical CO_2_ (SC-CO_2_) with full factorial design to determine the optimum extraction parameters. Nine extracts were obtained by varying pressure, temperature, and time. The extracts were analysed using FTIR, UV-Vis, and GC-MS. Cytotoxicity of the extracts was evaluated on human (colorectal, breast, and prostate) cancer and normal fibroblast cells. Moderate pressure (31.1 MPa) and temperature (60°C) were recorded as optimum extraction conditions with high yield (1.74%) of the extract (B2) at 60 min extraction time. The optimized extract (B2) displayed selective cytotoxicity against prostate cancer (PC3) cells (IC_50_ 28 *µ*g/mL) and significant antioxidant activity (IC_50_ 42.8 *µ*g/mL). Elevated levels of caspases 3/7 and 9 in B2-treated PC3 cells suggest the induction of apoptosis through nuclear and mitochondrial pathways. Hoechst and rhodamine assays confirmed the nuclear condensation and disruption of mitochondrial membrane potential in the cells. B2 also demonstrated inhibitory effects on motility and colonies of PC3 cells at its subcytotoxic concentrations. It is noteworthy that B2 displayed negligible toxicity against the normal cells. Chemometric analysis revealed high content of essential oils, hydrocarbon, fatty acids, esters, and aromatic sesquiterpenes in B2. This study highlights the therapeutic potentials of SC-CO_2_ extract of cat's whiskers in targeting prostate carcinoma.

## 1. Introduction

Leaves of* Orthosiphon stamineus* Benth. (Lamiaceae) have been widely used as herbal tea called “cat's whiskers or Java tea” in Asian and European countries. The herb is widely distributed in Southeast Asian countries, particularly Malaysia, Indonesia, and China. Leaves of the herb have been traditionally used for the treatment of variety of ailments including cancer, arthritis, hypertension, and renal stones [1]. It is reported that the herb has dynamic pharmacological properties including anti-inflammatory, antioxidant, and antibacterial properties [2]. The extracts of* O. stamineus* have been shown to be exceptionally safe with no signs of toxicity* in vivo* [3]. Antitumour studies have shown that solvent extract of* O. stamineus* leaves inhibited human colon tumour in nude mouse xenograft model [4, 5]. Detailed chemical analysis has shown the presence of active principle such as rosmarinic acid, sinensetin, eupatorin, and 3′-hydroxy-5,6,7,4′-tetramethoxyflavone [5]. It is suggested that the* in vivo* antitumor efficacy of the solvent extracts could be due to the collective contribution of antioxidant-rich phytochemicals [4].

When dealing with the extraction of botanical products, the key issues for both researchers and pharmaceutical companies are the effect of the extraction process on the nutritional or active medicinal components, including their toxicity and the residues of solvent. Conventional extraction methods have some disadvantages such as the long extraction time, the inconsistent temperatures that affects the active compounds, and the use of chemical solvents and the residual solvents in the final product. Thus, nowadays supercritical fluid extraction (SFE) is found worthwhile in the extraction of natural products. Supercritical carbon dioxide (SC-CO_2_) is the best alternative method which employs CO_2_ as an extraction solvent. It is inexpensive and environment-friendly as it can be recycled. It is nontoxic and safe with no residual solvents in the extract [6]. The additional advantage of this technique is that it uses a low temperature for extraction which helps to prevent sample degradation [7]. Similarly, a number of studies have reported the advantages of SC-CO_2_ in extraction of active compounds from medicinal herbs [8].

To our knowledge, so far, there have been no reports on the effect of SC-CO_2_ on extraction yield and cytotoxicity of* O. stamineus* leaves extracts. Thus, in the present work, an attempt was made to optimize the different extraction conditions using supercritical fluid extraction method to obtain the maximum yield of the extract of* O. stamineus* leaves with potent antioxidant activity. The extracts were tested for antiproliferative property against a panel of human cancer cell lines. Further a series of investigations were conducted on prostate cancer cell line to elucidate the mechanism of action. Chemometric analysis of the SC-CO_2_ extracts was studied using UV, FTIR, and GC-MS to correlate the potential biological activity with the chemical composition.

## 2. Materials and Methods

### 2.1. Plant Material and Reagents

Leaves of* O. stamineus* were collected from the botanical garden, School of Pharmaceutical Sciences, Universiti Sains Malaysia (USM), in March 2013. The floral characteristics of* O. stamineus* were studied and confirmed by the Senior Botanist Mr. Shanmugan, School of Biological Sciences, USM. The herbarium specimen (Voucher number: 11009) was deposited at the herbarium of School of Biology, Universiti Sains Malaysia. Commercial liquid carbon dioxide gas with purity of 99 g kg^−1^ in a gas cylinder at a temperature below −5°C was supplied locally from Malaysian Oxygen Company, Penang, Malaysia. Analytical GC-grade n-hexane, absolute (99.9%) ethanol (Fisher Scientific, Loughborough, UK) and MTT (3-(4,5-dimethylthiazol-2-yl)-2,5 diphenyl tetrazolium bromide) were purchased from Sigma-Aldrich, Germany. DMSO (dimethyl sulfoxide) was obtained from Sigma-Aldrich, Germany. All chemicals used were either HPLC or analytical grade. Human caspases 3/7, caspase 8, and caspase 9 FAM FLICA kits and Hoechst 33342 and Rhodamine 123 stains were purchased from Immunochemistry Technologies (Minnesota, USA).

### 2.2. Cell Culture and Cell Lines

Human colorectal carcinoma (HCT 116), hormone sensitive and invasive breast cancer (MCF-7), hormone resistant breast cancer (MDA-MB-231), prostate carcinoma (PC-3), and human normal fibroblast (CCD-18Co) cell lines were obtained from American Type Culture Collection (ATCC), Rockville, MD, USA. Cells were cultured at 5% CO_2_-humidifed atmosphere at 37°C in growth medium supplemented with 10% heat-inactivated fetal bovine serum (HIFBS) and 1% penicillin/streptomycin (PS). HCT 116 and MCF7 cells were cultured in RPMI-1640 and MEM media, respectively. PC-3 and CCD-18Co cells were cultured in F-12K and MDA-MB-231 cells were grown in DMEM supplemented with 5% HIFBS and PS.

### 2.3. Supercritical Carbon Dioxide (SC-CO_2_) Extraction

The SC-CO_2_ extraction was conducted using a supercritical fluid extractor SFX 220 (ISCO, Inc., Lincoln, NE, USA; model SFX 220) with 2 mL extractor vessel capacity. The flow rates for liquid CO_2_ were fixed at 1.5 mL/min. All variables (extraction time, pressure temperature) and CO_2_ flow rates were adjusted. General factorial design of three variable multilevels was applied with thirty-six runs to investigate the operating parameters that influence the supercritical extraction condition of* O. stamineus* tea leaves to obtain the highest yield. One gram powder of* O. stamineus* leaves (500 *μ*m in diameter) was placed into the extractor vessel. The extraction was then performed under various experimental conditions in accordance with the full factorial design. The first independent variable studied was extraction pressure (MPa) at three various levels (20.7, 31.1, and 41.4 MPa). The second independent variable was temperature with three levels (40, 60, and 80°C). Time is the third variable with four levels (15, 30, 45, and 60 min). The yield was recorded at each design point. Extraction was carried out at all the design points and each extraction was run in three replicates. The extracts were collected and weighed to calculate the percentage yield and stored at −20°C until further use.

### 2.4. Characterization and Phytochemical Analysis

#### 2.4.1. FTIR

FTIR spectra were recorded at wavelength range from 4000 to 400 cm^−1^ using Thermo-Nicolet Nexus 670 spectrometer (Thermo Scientific, USA) equipped with OMNIC application software (Thermo, Electron Corporation, USA).

#### 2.4.2. UV-Vis Spectrophotometry

UV-Vis spectrophotometry was carried out using Lambda25 UV/Vis spectrophotometer system operated with UV WinLab V2.85 software (Perkin Elmer, USA). Samples were prepared in methanol at 100 *μ*g/mL and were scanned at the wavelength range from 500 to 200 nm.

#### 2.4.3. Gas Chromatography-Mass Spectroscopy (GC-MS) Analysis

Quantitative chemical analysis of the SC-CO_2_ extracts was carried out using GC-MS with an aim to characterize the compound or group of compounds that might be responsible for the observed proapoptotic activity. The assay conditions were as follows: HP-5MS capillary column (30 m × 0.25 mm ID × 0.25 *μ*m, film thickness); held at 70°C for 2 min, raised to 285°C at a rate of 20°C/minute, and held for 20 min; 285°C for MSD transfer line heater; carrier helium at a flow rate of 1.2 mL/minute; 2 : 1 split ratio. 1 *μ*L solution of the extract in chloroform (10 mg/mL) was injected automatically. Scan parameter low mass: 35 and higher mass: 550. The constituents were identified by comparison with standards using NIST 02. A total ion chromatogram (TIC) was used to compute the percentage of the identified constitutes.

### 2.5. Assessment of Antioxidant Activities

#### 2.5.1. DPPH Radical Scavenging Activity

DPPH (1,1-diphenyl-2-picrylhydrazyl) assay [9] was carried out to evaluate the scavenging activity of SC-CO_2_ extracts.

#### 2.5.2. ABTS Assay

Free radical scavenging capability of the extracts was assessed using ABTS assay following the method described by Khadeer Ahamed and coworkers [9].

### 2.6. In Vitro Anticancer Studies

#### 2.6.1. Antiproliferation MTT Assay

MTT assay [10] was performed to assess the cytotoxicity of the SFE extracts on various cancer cell lines (HCT 116, MCF 7, PC-3, and MDA-MB-231). Human colonic fibroblast (CCD-18Co) was used as the model cell line for normal cell. The assay plates were read using Tecan microplate reader (Infinite M 200 PRO) at 570 nm absorbance. DMSO (0.1%) was used as a negative control.

#### 2.6.2. Cell Migration Assay

For cell migration assay [11], PC3 cells were seeded and incubated for 48 h to achieve fully confluent (90–100%) monolayer.

#### 2.6.3. Colony Formation Assay

Effect of B2 on clonogenicity of PC3 cells was investigated by colony formation assay [12]. The anticlonogenicity of the B2 was estimated in the form of percentage of plating efficiency (PE%) and percentage of surviving fraction (SF%).

#### 2.6.4. Determination of Nuclear Condensation by Hoechst 33342 Stain

Effect of B2 on nuclear chromatin condensation in PC3 cells was quantified by fluorescence microscopy using Hoechst 33258 stain [13].

#### 2.6.5. Detection of Mitochondrial Membrane Potential by Rhodamine 123 Stain

Detection of the changes in mitochondrial membrane potential in PC3 cells treated with B2 was assessed by the retention of rhodamine 123 [14].

#### 2.6.6. Caspase Inhibition Assay

The activity of caspases 3/7, caspase 8, and caspase 9 in PC3 cells treated with B2 was detected using the carboxyfluorescein FLICA apoptosis detection kit [15] following the manufacturer's instructions (Immunochemistry Technologies, LLC). The percent of caspase induction (CI) was calculated.

### 2.7. Statistical Analysis

All the results were subjected to ANOVA tests. The responses, the yields, the antioxidant activity, and the proliferative activity of the SC-CO_2_ extractions were compared at 5% significance level. The statistical analysis was conducted using Minitab 16 software (Minitab Inc., State College, PA, USA).

## 3. Results

### 3.1. Effect of SC-CO_2_ Extraction Conditions on* O. stamineus* Extracts

**Figure 1:**
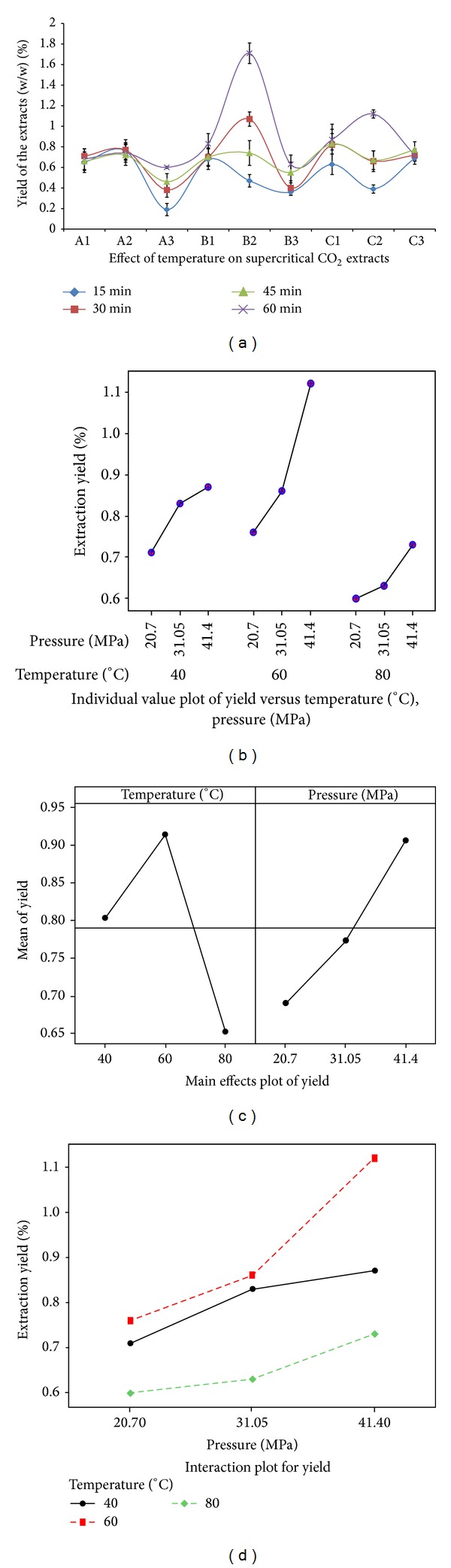
Effect of temperature, pressure, and time on the yield of SC-CO_2_ extracts of* O. stamineus*. (a) Percentage yield (g 100 g^−1^) of the 9 SC-CO_2_ extracts of* O. stamineus* leaves as a function of 4 different time periods (15, 30, 45, and 60 min). The graph shows that 60 min is the optimum time of extraction to obtain high yield. (b) Percentage yield (g 100 g^−1^) of the 9 SC-CO_2_ extracts of* O. stamineus* leaves with respect to the interaction of temperature and pressure. (c) Graphical representation showing the opposite correlation of temperature and pressure with the percentage yield. The pressure showed positive influence on the yield whereas the temperature showed negative impact on yield of the extracts. (d) Graphical illustration of the effect of different pressures at fixed temperatures. The figure reveals that as the pressure increased the percentage yield of the extracts also increased at all tested temperatures.

Effects of extract time, temperature, and pressure were studied on the yield and biological activity of the extracts. Cumulative percentage yield of SC-CO_2_ extracts of* O. stamineus* leaves was calculated at intervals of 15 min. The percentage yield increased significantly with increasing extraction time (*P* < 0.05). The results indicate that 60 min is the optimum extraction time for maximum yield of the extract of* O. stamineus* leaves by SC-CO_2_ (Figure 1(a)).

Cumulative percentage yield of the extracts was estimated at intervals of 20°C and approximate 10 MPa pressure.  Figure 1(b) shows the interaction between temperature and pressure. The extraction temperature showed a negative correlation with the percentage yield at 40, 60, and 80°C with decreasing order of *R* ^2^, respectively, whereas the extraction pressure showed significantly (*P* < 0.05) positive correlation with the percentage yield at 20.7, 31.1, and 41.4 MPa with *R* ^2^ 0.92, 0.95, and 0.96, respectively (Table 1).  Figure 1(c) shows the behavior of two factors (pressure and temperature) separately and their effect on the extraction yield. It can be seen that the two factors have significant effect on the extraction yield. The results of ANOVA analysis exhibited that the effect of these parameters was significant on yield of extraction *P* < 0.05.

The results showed that the pressure increment has enhanced the extraction yield at all tested temperatures (Figure 1(d)). At a fixed extraction temperature of 40°C, increasing the pressure from 20.7 to 41.4 MPa has increased the extraction yield from 0.71 to 0. 87%, respectively. However, it was found that the extraction rate was enhanced 47% at extraction temperature of 60°C. Figure 1(d) depicts that the extraction yield increased significantly from 0.76 to 1.74% with increasing pressure from 20.7 to 41.4 MPa, respectively. Likewise, at a fixed extraction temperature of 80°C, the extraction yield increased from 0.6% to 0.73 by increasing the pressure from 20.7 to 41.4 MPa, respectively. However, operating extraction pressure of 20.7 MPa was not very beneficial for the extraction yield. As at this extraction pressure the extraction rate at temperature of 40°C was 22.5%, which then increased to 47% at extraction temperature of 60°C and then abruptly dropped to 21.6% at temperature of 80°C. Altogether, the highest extracted yield was 1.74% (B2 extract) which was obtained at extraction pressure of 31.1 MPa, using extraction temperature of 60°C, during a period of 60 min as shown in Table 1.

### 3.2. Phytochemical Study of the Extracts

#### 3.2.1. FTIR Analysis

**Figure 2:**
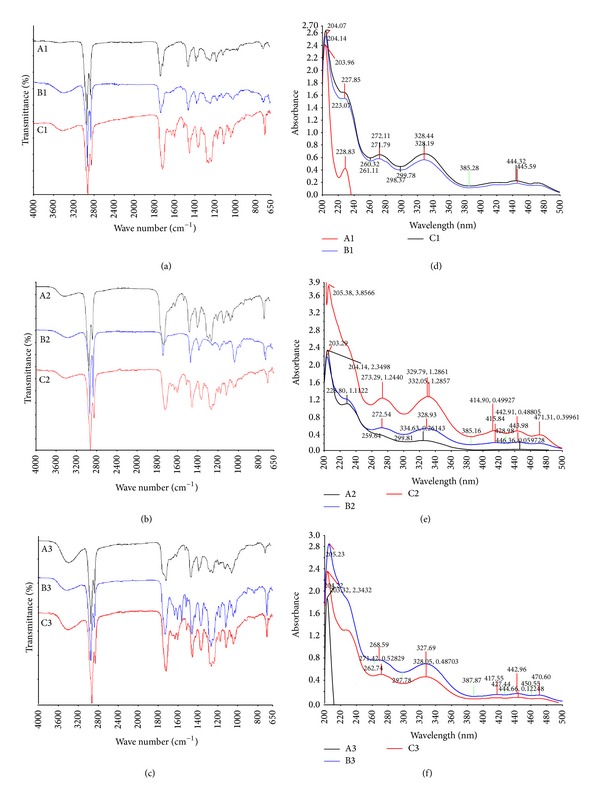
FTIR and UV-Vis spectra of the 9 SC-CO_2_ extracts of* O. stamineus*. (a) The extracts A1, B1, and C1 (40°C). (b) The extracts A2, B2, and C2 (60°C). (c) The extracts A3, B3, and C3 (80°C). (d) The extracts A1, B1, and C1 (40°C). (e) The extracts A2, B2, and C2 (60°C). (f) The extracts A3, B3, and C3 (80°C).

FTIR spectra of the extracts obtained at 60 min are shown in Figures 2(a)–2(c).  Table 2 depicts the related functional groups of transmittance bands of the corresponding wavelengths [16].

#### 3.2.2. UV-Vis Spectrophotometry

The UV spectra for the extracts A1, B1, and C1 (Figure 2(d)) displayed strong absorption bands at *λ*_max⁡_ 228–232 nm, the characteristic feature for a conjugated double bond, whereas the other extracts showed feeble absorption around this region (Figures 2(e) and 2(f)). Spectrum of the extract A3 showed that the extract lacks the *π*-electrons of a double bond or a benzene ring shifts. Further the UV spectra of all extracts, except A1 and A3, showed obvious bands at *λ*_max⁡_ 328, 279, 213, 337, 272, 205, 332, 273, and 228 nm.

### 3.3. Antioxidant Activity of the SC-CO_2_ Extracts of* O. stamineus* Leaves

DPPH and ABTS radicals scavenging activity is presented in terms of the IC_50_ in Table 3. It was found that the extracts produced at moderate temperatures and higher pressure (B2 and C2) showed significant antioxidant activity compared to the other extracts. The extract C2 produced at 60°C and 41.4 MPa showed significant (*P* < 0.01) DPPH quenching activity (IC_50_ = 56.1 *μ*g/mL). However, the extract B2 (60°C and 31.1 MPa) demonstrated more pronounced antioxidant activity than the other tested extracts. The IC_50_ values of B2 were 91.7 and 42.8 *μ*g/mL for ABT and DPPH, respectively (*P* < 0.05).

### 3.4. Antiproliferative Activity of SC-CO_2_ Extracts of* O. stamineus* Leaves

**Figure 3:**
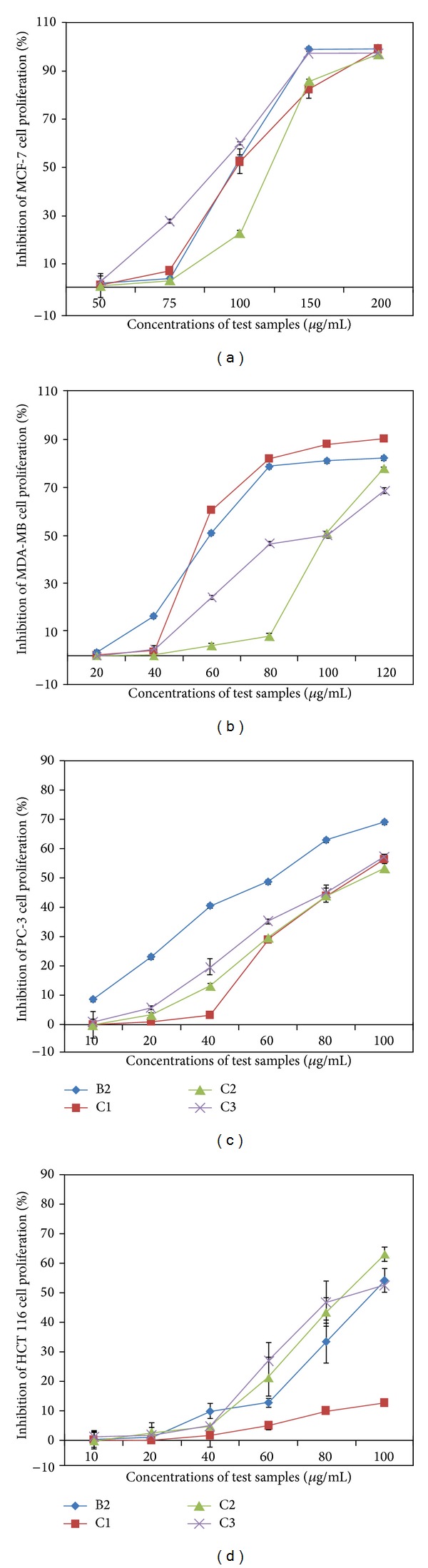
Dose dependent antiproliferative effect of SC-CO_2_ extracts of* O. stamineus*. (a) The antiproliferative effect of B2, C1, C2, and C3 on estrogen-dependent human breast cancer (MCF7) cells. (b) The antiproliferative effect of B2, C1, C2, and C3 on estrogen-independent human breast cancer (MDA-MB 231) cells. (c) The antiproliferative effect of B2, C1, C2, and C3 on human prostate cancer (PC-3) cells. (d) The antiproliferative effect of B2, C1, C2, and C3 on human colorectal carcinoma (HCT 116) cells.

**Figure 4:**
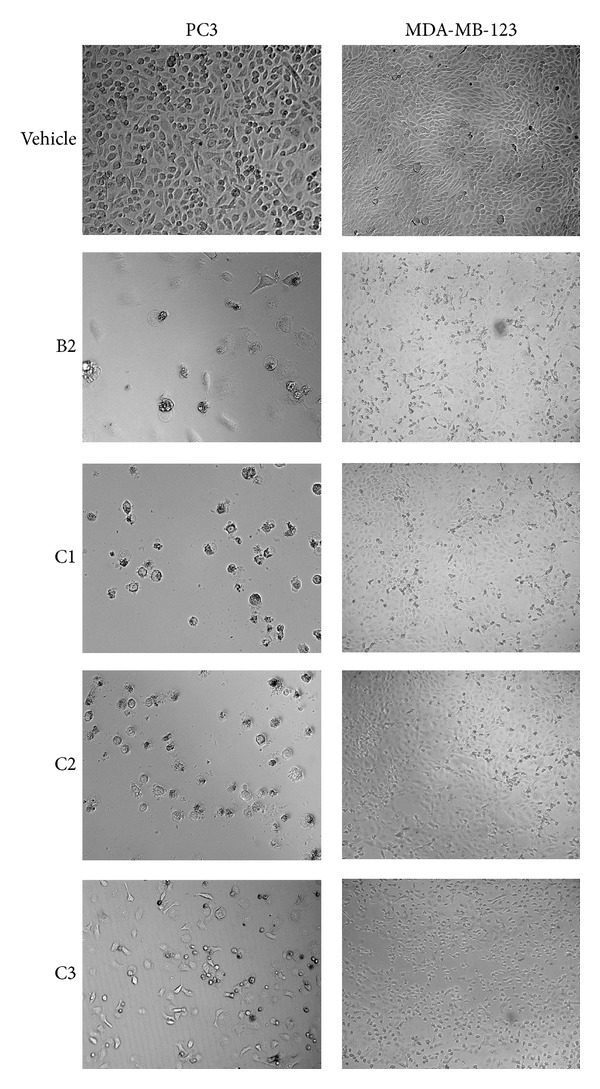
Effect of cytotoxicity of SC-CO_2_ extracts of* O. stamineus*. Photomicrographic images of PC3 and MDA-MB-231 cells taken under an EVOS f1 digital microscope at 20x magnification after 48 hours of treatment with the B2. The vehicle (0.1% DMSO) treated cells showed fully confluent growth with compact layer of highly proliferating HCT 116 cells. The cells displayed well established pseudopodial cellular projections with prominent nuclei, whereas the extracts (B2, C1, C2, and C3) treated cells demonstrated remarkable inhibitory effect on proliferation of both cell types. The pictures revealed that the population of cells reduced drastically within the 48 hours of treatment. In addition, the extracts induced noticeably morphological changes in the cells.

Antiproliferative effect of different SC-CO_2_ extracts of* O. stamineus* on various human cell lines was tested using MTT assay. Similar to the antioxidant activities, significant (*P* < 0.01) antiproliferative activities have been shown from the extracts obtained under medium and high pressures. The IC_50_ values of the SFE extracts of* O. stamineus* on various human cell lines are shown in Table 4. The extracts (B2, C1, C2, and C3) prepared at high pressures demonstrated considerable cytotoxic activity against PC3 cell line. The IC_50_ values of C1, C2, and C3 were 79, 78, and 73 *μ*g/mL, respectively, on PC3 cells. However, among all the extracts, B2 displayed more pronounced antiproliferative effect against PC3 with IC_50_ 28 *μ*g/mL. In addition, B2 and C1 exhibited significant (*P* < 0.05) antiproliferative effect against breast (MDA-MB-231) cell line with IC_50_ 57.8 and 53 *μ*g/mL, respectively (Table 3).  Figure 3 shows the dose-dependent antiproliferative effects of B2, C1, C2, and C3 on MCF7 (Figure 3(a)), MDA-MB-231 (Figure 3(b)), PC-3 (Figure 3(c)), and HCT 116 (Figure 3(d)) cell lines.  Figure 4 shows that the photomicrographic images of the treated PC3 and MDA-MB-231 cells presented clear evidence of significant cytotoxicity of the extracts, as the vehicle (0.1% DMSO) treated cells displayed a compact monolayer of aggressively growing cancer cells with prominent nuclei and intact cell membrane, whereas the images taken form the extracts treated group showed a drastic reduction in the number of cells because of the antiproliferative activity of the extracts. In addition, the extracts severely affected the pseudopodial projections of the cells which rendered the cells non-adherent and become round shaped. Interestingly, all the extracts studied showed either mild or negligible cytotoxicity towards the CCD-18Co cell line which was used as a model cell line for the normal human cells.

Overall result of cytotoxic test on the human cancer cell lines displayed that the extract B2 demonstrated potent cytotoxicity selectively towards the PC3 cell line as the IC_50_ obtained is much lower than that of other cell lines. Therefore, in the present study, PC3 cell line is selected to study the effect of B2 on the motility, clonogenicity, morphological modifications, nuclear condensation, mitochondrial membrane potential, and induction of caspases in PC3 cells.

### 3.5. Inhibitory Effect of B2 on Migration and Clonogenicity of PC3 Cells

**Figure 5:**
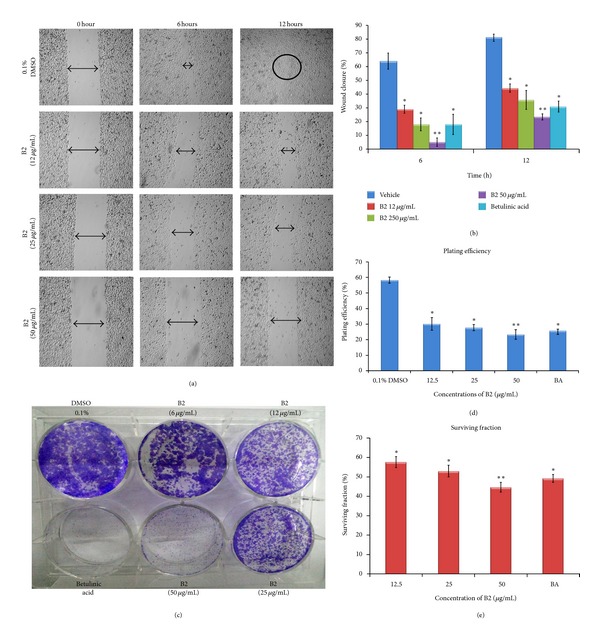
Effect of B2 on migration and colony formation of PC3 cells. For migration assay, a straight scratch wound was created using a 200 *μ*L micropipette tip. The cells were treated with B2 (12, 25, and 50 *μ*g/mL). DMSO (0.1%) and betulinic acid were used as negative and positive controls, respectively. The wounds were photographed using EVOS f1 digital microscope at zero, 6, and 12 h. (a) The images taken form 0.1% DMSO treated cells showed completely closed wound due to the successful migration of PC3 cells within 12 h, whereas in B2-treated group, the wound remained open even after 12 h period time. B2 (12 *μ*g/mL) caused significant inhibition of PC3 cell migration at subcytotoxic dose. (b) Graph illustrates the time- and dose-dependent inhibitory effect of B2 on migration of PC3 cells. The distance of cell-free area was measured using Leica Quin software, and the results are presented as mean percentage of migration inhibition ± SD in comparison with the negative control. (c) Inhibitory effect of B2 on survival of PC3 colonies in colony formation assay. The picture clearly depicts the dose-dependent inhibition of PC3 colonies. PC3 cells (500 cells/well) were seeded in 6-well plate and treated with B2 (6, 12, 25, and 50 *μ*g/mL) for 48 h. Betulinic acid (10 *μ*g/mL) and 1% DMSO were used as positive and negative controls, respectively. The cells were maintained until sufficiently large colonies (≥50 cells) were produced for 10 days. The colonies were fixed, stained with 0.2% crystal violet, and counted under stereomicroscope. (d) Graphical representation depicts percentage of plating efficiency of representative test groups. Plating efficiency was determined by the percent ratio of number of colonies developed to the number of cells initially seeded. (e) Graphical representation illustrates the percentage of surviving fraction obtained after the treatment with B2. The percent surviving fraction of the PC3 colonies was decreased with increasing concentration B2. All the values are presented as mean ± SD (*n *= either 6 or 10), ∗ represents *P* < 0.05, and ∗∗ represents *P* < 0.01.

B2 demonstrated dose- and time-dependent inhibitory effect on migration of PC3 cells. B2 displayed significant inhibition in cell motility (Figure 5(a)) at the concentration lower than its IC_50_ on PC3 cells. The percentage of wound closure calculated after 12 h was 29 ± 3, 18 ± 5, and 5 ± 3% at 12, 25, and 50 *μ*g/mL (*P* < 0.05). Even after 12 h the percentage of wound closure was significantly inhibited by B2. Figure 5(b) graphically illustrates the potent inhibitory effect of B2 on motility of PC3 cells which can be compared with the standard reference, betulinic acid.

The dose-dependent inhibitory effect of B2 on colony formation of PC3 cells was depicted in Figure 5(c). Percentage of plating efficiency (PE) in vehicle (0.1% DMSO) treated cells was 58 ± 2% which was drastically decreased to 30 ± 4, 28 ± 2, and 23 ± 3% upon treatment with B2 at 12, 25, and 50 *μ*g/mL, respectively (Figure 5(d)). The surviving fraction (SF) was determined to be 58 ± 3, 53 ± 3, and 45 ± 2% after B2 treatment at the concentrations 12, 25, and 50 *μ*g/mL, respectively (Figure 5(e)). These results can be compared with the standard reference, betulinic acid.

### 3.6. B2 Induces Morphological Modifications and Nuclear Condensation in PC3 Cells

**Figure 6:**
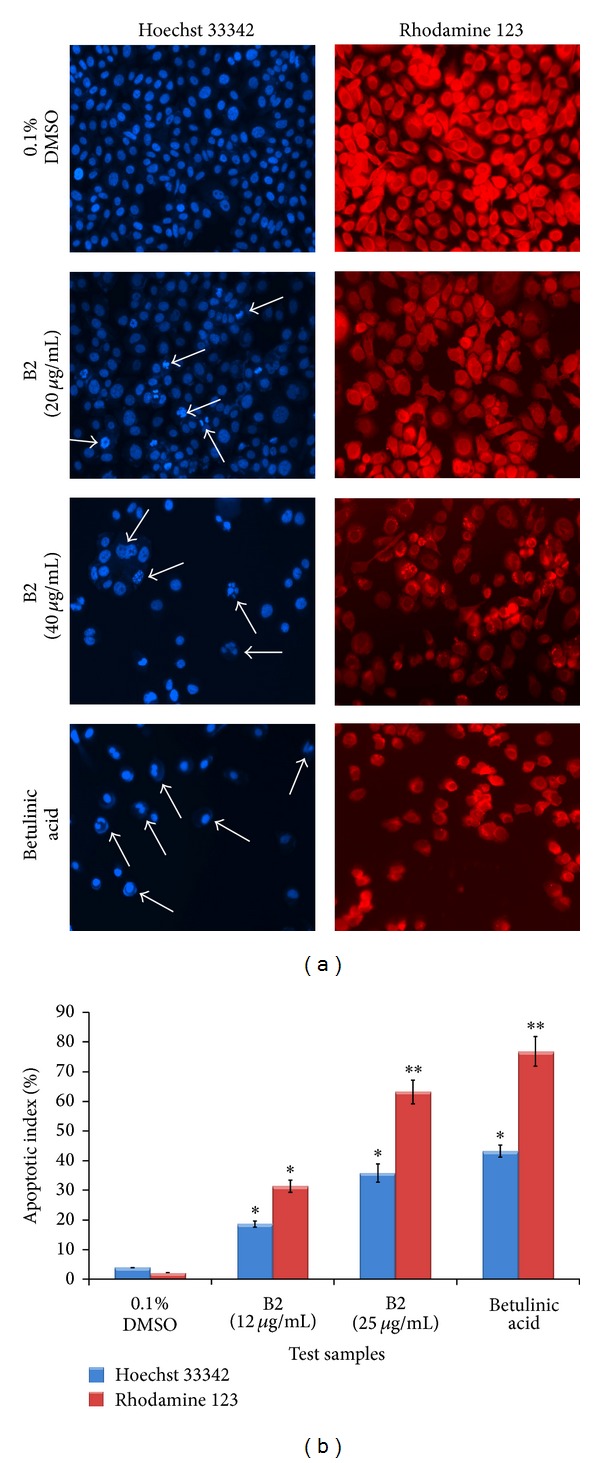
Proapoptotic effect of B2 on PC3 cells. The cells were treated with two concentrations (12 and 25 *μ*g/mL) of B2 and analysed separately at 12 hr. DMSO (0.1%) and betulinic acid (10 *μ*g/mL) were used as negative and positive controls, respectively. The cells were fixed in 4% paraformaldehyde and then stained with either Hoechst stain 33342 (1 *μ*g/mL in PBS) or rhodamine 123 (5 *μ*g/mL) for 20 min. The cellular morphology was photographed at 20x magnification, using EVOS f1 digital microscope (Advanced Microscopy Group, USA). (a) The photomicrographs in the left column depict the images of PC3 cells stained with Hoechst 33258. The cells treated with 0.1% DMSO (negative control) appeared as confluent monolayer with prominent nuclear and other cellular features. The cells treated with B2 displayed clear characteristic changes of apoptosis. The arrows showed the condensed, fragmented, and crescent shaped nuclei indicating the early phase of apoptosis. In addition, the higher concentration of B2 displayed the chromatin dissolution, breakdown, and fragmentation (arrows), which imply the advanced stage of apoptosis. The results can be compared to that of the standard reference, betulinic acid. The photomicrographs in the right column depict the images of PC3 cells stained with rhodamine 123. The images provide the clear evidence of disruption in the mitochondrial membrane potential caused by B2. The effect in PC3 cells was assessed by visualizing the rhodamine 123 fluorescence signals in the mitochondria. Treatment of cells with B2 showed that the signal decreased proportionally with respect to the decrease of mitochondrial membrane potential. (b) Graphical representation of percentage of apoptotic indices. The apoptotic index for each test group was expressed as a percentage of the ratio of apoptotic cells number to the total cell number in 10 different fields. In Hoechst stain assay, cells with densely blue-colored, condensed, or fragmented nuclei were considered apoptotic. The number of cells with apoptotic morphology was counted in randomly selected fields per well. The apoptotic index was calculated as a ratio of apoptotic cells to the total number of cells and presented as the mean ± SD (*n* = 10); ∗ represents *P* < 0.05 and ∗∗ represents *P* < 0.01.

In the present study, the effect of B2 on PC3 cells was tested to study morphological modifications and nuclear condensation using Hoechst 33342 stain. Microscopic examination revealed that B2 induced the typical apoptotic morphological changes in PC3 cells in a dose-dependent fashion (Figure 6(a)). Nevertheless, the cells from vehicle (0.1% DMSO) group demonstrated regular architecture of monolayer of PC3 cells with prominent nuclei and intact cell membrane. After 12 hr of B2 treatment, the condensation of nucleus and dissolution of chromatin can be seen clearly in the cytoplasm. At high concentration of B2 (40 *μ*g/mL), the cells displayed shrunken and crescent shaped nuclei with discrete chromatin bodies which are the characteristic features of advanced stage of apoptosis. The percentage of apoptosis index estimated for negative control was 4 ± 0.02% which was significantly increased after the treatment (Figure 6(b)). However, the apoptotic indices for the cells treated with B2 were significantly increased to 19 ± 1 and 36 ± 3% at 12 and 25 *μ*g/mL concentrations, respectively (Figure 6(b)). Positive control group treated with betulinic acid (10 *μ*g/mL) showed remarkable apoptosis with 43% apoptotic index.

### 3.7. Treatment of B2 Reduced Mitochondrial Membrane Potential in PC3 Cells

The cells were exposed to rhodamine 123 after 12 hr of treatment and the intensity of rhodamine in the cells was observed (Figure 6(a)). Results of the present study showed an obvious intensification of fluorescence in the untreated cells; however, the signal significantly (*P* < 0.01) dropped in the cells treated with B2, which proposes the damage in mitochondrial membrane and decrease in membrane potential. The dose-dependent effect was evident from the apoptotic indices calculated which were 31 ± 2 and 63 ± 4% after the treatment with B2 at 12 and 25 *μ*g/mL, respectively (Figure 6(b)). The result can be compared to that of betulinic acid (10 *μ*g/mL) which caused 77 ± 5% apoptotic index.

### 3.8. Inhibitory Effect of B2 on Caspases

**Figure 7:**
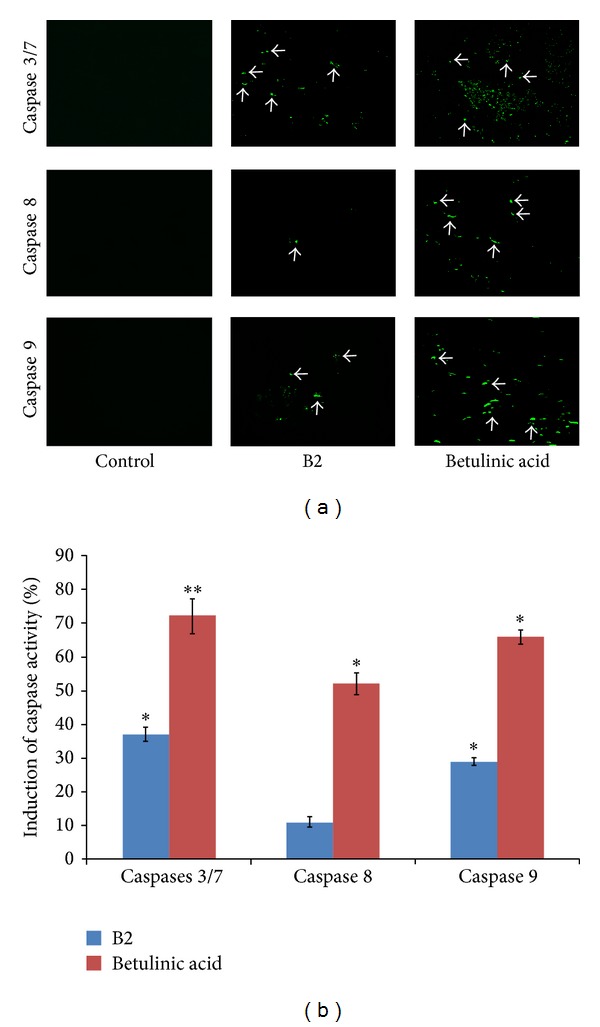
In caspase assay, PC3 cells (1 × 10^5^ cells/mL) were treated with subtoxic concentration (10 *μ*g/mL) of B2. Betulinic acid (10 *μ*g/mL) and DMSO (0.1%) were used as positive and negative controls. The cells were labeled with fluorescent FAM-VAD-FMK dye after 8 hours of treatment and incubated for 60 minutes. The media were removed thereafter and the cells were incubated in fresh media for another 60 minutes. The plate was centrifuged at 300 RPM for 5 minutes to include the detached apoptotic cells in the assay. Then the media were replaced by PBS in each well. The fluorescence intensity was recorded at 490 nm excitation and 520 nm emission using a fluorescence microplate reader (Multiskan Ascent micro plate reader, Thermolab system 354, Finland). The apoptotic cells were observed under fluorescent microscope at 20x magnification. (a) Photomicrograph of the fluorescence emitting cells. The images were taken by EVOS fluorescence microscope at 20x magnification. (b) Graphical representation depicts the quantitative estimation of percent induction of caspase activity in PC3 cells by B2 and betulinic acid (BA).

In the present study, the activity of caspases 3/7, caspase 8, and caspase 9 was detected in B2-treated PC3 cells by fluorescence microplate reader and fluorescence microscopy using the carboxyfluorescein FLICA apoptosis detection kit. The fluorescent intensity in the photomicrographic images of the PC3 cells (Figure 7(a)) shows the induction of caspase activities. The results of the present study demonstrated that B2 caused significant induction of caspases 3/7 and caspase 9 activities. In addition, considerable activation of caspases 8 was also detected (Figure 7(a)).  Figure 7(b) illustrates the quantitative estimation of caspase induction caused by B2 in comparison with positive control, betulinic acid.

### 3.9. GC-MS Analytical Report

**Figure 8:**
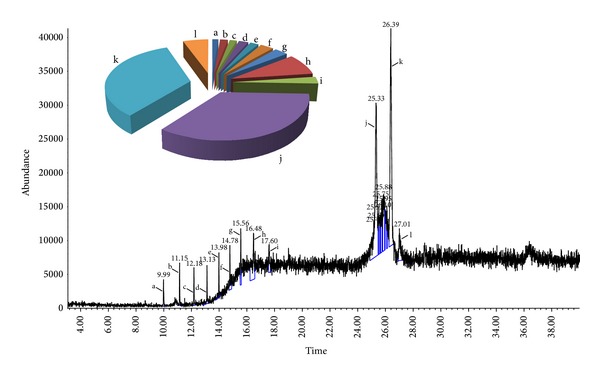
Chemical characterization of the extract B2 on mass spectrum of GC-MS was conducted with the help of the Metabolites Spectral Database and NIST (National Institute of Standards and Technology) Library (Ezhilan and Neelamegam, 2012). The identification of the major chemical components was based on similarity index (SI), Wiley 8 computer library. The mass spectrum of the unknown component was compared with the spectrum of the known components stored in the library. The retention time, nomenclature, molecular weight, structure, and composition of the major components were recorded. The pie charts depict the relative chemical compositions of subfractions. The chart illustrates that the dominant peaks of B2 are acetic acid (3-hydroxy-7-isopropenyl-1,4a-dimethyl-octahydronaphthalen-2-yl) ester (27.01%), dimethyl-4-(1-methylethylidene)-2,4,6,7,8,8a-hexahydro-5(1H)-azulenone (26.86%), and anthracene, 9-(2-propenyl) (25.33%). The details of the peaks are given in Table 1.

The extract B2 was analysed using GC-MS to specify and quantify the major chemical constituents present in it.  Figure 8 depicts the GC-MS of B2. The pie chart in Figure 8 illustrates the composition and proportion of the major chemical constituents in the extract. Detailed characteristics of the peaks identified in the GC-MS (Figure 8) were presented in Table 5.

## 4. Discussion

Supercritical carbon dioxide (SC-CO_2_) extraction method is an attractive and more advanced technology than the conventional solvent extraction methods. SC-CO_2_ is nonexplosive, nontoxic, inexpensive, and able to solubilize lipophilic substances [17, 18]. Carbon dioxide has been shown to ensure minimal alteration of the bioactive compounds and thereby it preserves the native chemical properties of the compounds and thus the curative and functional properties of the compounds will be retained [19]. Therefore, SC-CO_2_ extraction method gives the extracts with better biological activity than the conventional extractions methods. Moreover, CO_2_ is gaseous at room temperature and pressure, which makes the phytochemicals recovery very easy and simple and provides solvent-free extracts. In addition, CO_2_ is environment-friendly and generally recognized as safe by the Food and Drug Administration and European Food Safety Authority.

In the present study, a full factorial design of 3 × 3 × 4 was carried out with total number of thirty six runs in order to determine the best extraction conditions in terms of extraction yield and antiproliferative and antioxidant properties. The findings of the present study revealed that the percentage yield of the extract was time and pressure dependent and thus the percentage yield increased significantly with increasing pressure and time (*P* < 0.05). In contrast, the percentage yield was noted to be temperature independent. However, the extraction yield was increased with increasing pressure at a fixed extraction temperature. This increment is due to the fact that high pressure increases the density of a supercritical fluid that consequently enhances the solubilizing capability of organic compounds and ultimately helps in dissolving large quantities of the phytoconstituents [20]. On the other hand, temperature poses an opposite effect on the density of SC-CO_2_ and therefore the density of SC-CO_2_ decreases with increasing extraction temperature at a given operating pressure. This results in reduced solvating power of CO_2_ which then affects the solubility of the compounds and decreases the extraction efficiency [20]. This could be the reason for the variant effects of temperature on the extraction yield.

Analysis of UV-Vis spectroscopic data indicates a highly polyene nature of the phytoconstituents. Particularly, the extracts A2, B1, B2, B3, C1, C2, and C3 showed clear characteristic peaks for prenyl flavone skeleton compounds such as sinensetin, eupatorin, and tetramethoxyflavone, which are the characteristic markers of* O. stamineus*. FTIR spectral analysis of the extracts obtained from high temperatures revealed the prominent and broad signals which correspond to the hydroxyl groups. This clearly indicates that the extracts are rich in hydroxyl groups. Usually, polar compounds possess at least one terminal hydroxyl (–OH) group which makes them hydrophilic and therefore less volatile. On the other hand, the extracts obtained at moderate pressure (31.1 MPa) showed IR absorption frequencies corresponding to the olefinic carbon double bonds, which indicates the abundance of unsaturated phytochemicals such as phenolics, flavonoids, and other aromatic compounds.

The findings of the cytotoxic activity showed that, out of 9 extracts studied, B2 (prepared at 31.1 MPa and 60°C) showed selective antiproliferation activity against prostate cancer (PC3) cells. Interestingly, either negligible or insignificant cytotoxic effect of B2 was recorded on other tested cancer cells including the normal CCD-18Co cells. The cytotoxic effect of the B2 against the cancer cells could probably be due to its antioxidant-rich polyphenolic contents. This was further supported by the results of the antioxidant assays. In the present study, the extract B2 demonstrated noticeable scavenging activity against both the tested free radicals, ABTS and DPPH. The characteristic chemical markers of* O. stamineus* such as caffeic acid derivatives, polymethoxylated flavonoids, terpenes, and phenolics are well studied antioxidant compounds with potent cytotoxicity [21]. It has been reported that the abundant presence of methoxylated flavonoids (eupatorin and sinensetin) in* O. stamineus* imparts the cytotoxic property to the extract. Eupatorin was reported as a potent inhibitor for* in vitro* proliferation of breast cancer cells [22], while sinensetin was reported to inhibit the growth of gastric cancer cells and induces apoptosis [23].

In order to characterise the cytotoxicity induced by B2 in PC3 cells, a series of assays were conducted to get deeper insights into its mode of action. During tumorigenesis, the tumor cells continue their proliferation and migration into the secondary site which leads to metastasis. This is based on the migration and colonization of the tumor cells [24]. B2 suppressed colonization of PC3 cells in a dose-dependent manner. It is noteworthy that B2 demonstrated cytotoxic effect at high concentration (50 *μ*g/mL) and caused irreversible damage to the cell integrity; however, at low concentration (12 *μ*g/mL) B2 displayed cytostatic effect. The anticlonogenic results indicated that B2 is cytotoxic at high concentration while cytostatic at its low concentration, as evident by decrease of survival fraction. Colonization and migration are the critical processes to promote tumorigenesis and metastasis. In this study, inhibition of migration and colony formation of PC3 cells* in vitro* by B2 suggests that the extract could probably inhibit cell metastasis [25]. This indicates that the extract has potential to block the motility of the prostate cancer cells.

Apoptotic cells undergo a series of characteristic morphological and biochemical changes, which usually manifest themselves as nuclear condensation, DNA fragmentation, and chromatin dissolution [13]. In this study, Hoechst-stained images of B2-treated PC3 cells revealed clear proapoptotic signs such as chromatin condensation and dissolution. High concentration of B2 rendered the cells with crescent shaped nuclei, a characteristic feature of advanced stage of apoptosis.

To confirm the apoptosis induced by B2 in PC3 cells involved the loss of mitochondrial membrane potential and integrity in the cells, the effect was assessed by visualizing the uptake of rhodamine 123 by mitochondria. Rhodamine 123 is the lipophilic dye which can be readily absorbed into the mitochondria of live cells [26]. Disturbance in mitochondrial membrane potential (ΔΨ) is a clear indication of early apoptotic stage [27]. Uptake of rhodamine 123 by the cells decreases exponentially with decrease of mitochondrial membrane potential, and consequently the intensity of florescent also decreases. The photomicrographic images of B2-treated PC3 cells revealed remarkable reduction in mitochondrial membrane potential, a sign of apoptosis induction.

The proapoptotic effect of B2 on DNA and mitochondria was further confirmed by measuring the caspases activity in PC3 cells. Caspases are a group of intracellular cysteine proteases that promote apoptosis in the cells. Caspase 9 is an initiator caspase activated by mitochondria via intrinsic pathway. After getting activated, caspase 9 subsequently activates a series of “effector caspases,” caspases 3/7, and ultimately the cascade ends with cellular death [28]. However, caspase 8 is an executioner caspase and activated by extrinsic pathway.

The overall result demonstrates that the extract B2 at subcytotoxic concentration (10 *μ*g/mL) caused apoptosis which appears to be chiefly associated with the early activation of caspase 9 through mitochondrial pathway, as confirmed by the loss of mitochondrial membrane potential in Rhodamine 123 assay. The activation of the mitochondrial pathway may in turn induce caspase 8, eventually resulting in a feedback amplification of caspase 9. Caspases 3 and 7 are the executioner enzymes and their activation results into the nuclear condensation and degradation which further supports the findings of Hoechst stain assay on nuclear condensation and dissolution.

The proapoptotic effect of B2 was further supported by the GC-MS analysis. The major chemical constituents present in the extract were identified using the NIST library. Predominantly, the spectra of B2 showed presence of several biologically dynamic phytoconstituents such as the derivatives of anthracene, hydronaphthalen, and azulenone. Among the identified compounds, anthracene derivatives were reported to possess promising antitumor activity by binding, intercalating, and fragmenting DNA in cancer cells [29–31]. However, the other major compounds identified in B2 such as acetic acid (3-hydroxy-7-isopropenyl-1,4a-dimethyl-octahydronaphthalen-2-yl) ester and dimethyl-4-(1-methylethylidene)-2,4,6,7,8,8a-hexahydro-5(1H)-azulenone were reported as the major chemical composition of the extracts with considerable biological activities including cytotoxic properties [32, 33].

## 5. Conclusion

In the present study, a general factorial experiment of type 3 × 3 × 4 was used with total number of thirty-six runs to prepare supercritical CO_2_ (SC-CO_2_) extracts of cat's whiskers tea (*Orthosiphon stamineus*). The SC-CO_2_ extract prepared at moderate pressure and temperature (31.1 MPa and 60°C) produced selective cytotoxicity against human prostate cancer (PC3) cells and significant free radical scavenging activity, while being nontoxic to normal cells. Further investigation provided good evidence that the extract displayed proapoptotic, antimetastatic, and anticlonogenic effects in PC3 cells, which could be due to the collective contribution of phytochemicals, particularly, aldehyde, hydrocarbon, ketone, fatty acids, esters, and aromatic sesquiterpenes such as acetic acid (3-hydroxy-7-isopropenyl-1,4a-dimethyl-octahydronaphthalen-2-yl) ester, dimethyl-4-(1-methylethylidene)-2,4,6,7,8,8a-hexahydro-5(1H)-azulenone, anthracene, 9-(2-propenyl), 2-propen-1-one, 1,3-diphenyl, and 2-amino-2-oxo-acetic acid N-(3,4-dimethylphenyl) ethyl ester. Thus, the bioactive extract of cat's whiskers tea prepared using an inexpensive and environment-friendly supercritical CO_2_ could be a valuable bioresource with anticancer potential against prostate malignancy.

## Figures and Tables

**Table 1:** Percentage yield of the extracts obtained from *Orthosiphon stamineus* tea leaves using SC-CO_2_ in relation to time interval, temperature, and pressure.

Sample	Temperature	Pressure	Percentage yield (g/100 g) in the extraction time intervals				Correlation
code^*n*^	(°C)	(MPa)	15 min	30 min	45 min	60 min	*R* ^2^ value
A1	40	20.7	0.55 ± 0.1	0.66 ± 0.07	0.71 ± 0.07	0.66 ± 0.09	0.92
A2	60	20.7	0.42 ± 0.08∗	0.57 ± 0.1	0.68 ± 0.1	0.76 ± 0.1∗	0.79
A3	80	20.7	0.38 ± 0.06	0.42 ± 0.07	0.46 ± 0.07	0.46 ± 0.08	0.66
B1	40	31.1	0.48 ± 0.1	0.67 ± 0.09	0.70 ± 0.09	0.70 ± 0.08	0.95
B2	60	31.1	0.47 ± 0.06	1.07 ± 0.07∗	1.07 ± 0.07∗	1.74 ± 0.12∗	0.97
B3	80	31.1	0.36 ± 0.03	0.40 ± 0.07	0.40 ± 0.07	0.55 ± 0.1	0.89
C1	40	41.4	0.33 ± 0.1	0.74 ± 0.2	0.82 ± 0.2∗	0.87 ± 0.1∗	0.96
C2	60	41.4	0.32 ± 0.04	0.72 ± 0.1	0.66 ± 0.1	0.67 ± 0.09	0.93
C3	80	41.4	0.29 ± 0.06	0.66 ± 0.05	0.62 ± 0.05	0.64 ± 0.08	0.77

Results are presented as mean ± SD (*n* = 3); *P < 0.05.

**Table 2:** Transmittance bands correlated to the corresponding wave numbers representing the functional organic groups present in the SC-CO_2_ extracts of *Orthosiphon stamineus*.

Vibrational frequency (cm^−1^)	Samples	Corresponding organic groups
3600–3200 (broad)	A3, B3, and C3	O–H stretching
2950–2800	All nine samples	C–H stretching
1780–1720 (sharp)	All nine samples	C=O stretching
1660–1650 (weak)	C1, C2, B3 and C3	C=C stretching
1610–1600 (Sharp)	C1, A2, C2, A3, B3 and C3	NO_2_– stretching
1480–1460	All nine samples	C–H bending (deformation)
1385–1370	All nine samples	C–C, C–H, O–H and C–O bending
1240–1160	All nine samples except B2	C–C, C–O, C–N stretching
1100–1090	All nine samples except B2	C–C, C–O, C–N stretching
1050–1045	All nine samples except A1	C–C, C–O, C–N stretching
960–900	All nine samples	C–H bending
710–725	All nine samples	C–H bending

**Table 3:** Antioxidant activities of the extracts of* O. stamineus* leaves obtained by supercritical CO_2_.

Assays^a^	IC_50_ values^d^ of the samples in *μ*g/mL									
	A1	A2	A3	B1	B2	B3	C1	C2	C3	AA
ABTS^b^	ND	1150	ND	ND	91.7∗	ND	218	ND	117	6.5∗∗
DPPH^c^	>200	>200	>200	136	42.8∗	164	168	56.1∗∗	198	5.8∗∗

^a^Microtiter 96-well plate was used to assess the concentrations (12.5, 25, 50, 100, and 200 *μ*g/mL) of test samples. Methanol was used as blank. ABTS or DPPH was used as negative control. Ascorbic acid (AA) was used as reference standard.

^b^Absorbance for ABTS was measured at 734 nm.

^c^Absorbance for DPPH was measured at 517 nm.

^d^Using linear regression curves the median inhibitory concentrations (IC_50_) were calculated. The results are expressed as mean ± SD (*n* = 6); ∗P < 0.05; ∗∗P < 0.01.

**Table 4:** Antiproliferative properties of the extracts of* O. stamineus* leaves obtained by supercritical CO_2_.

Samples^a^	IC_50_ *μ*g/mL				
	MCF-7	MDA-MB	HCT116	PC3	CCD-18 Co
A1	>200	>200	>200	>200	>200
A2	>200	>200	>200	>200	>200
A3	>200	>200	>200	>200	>200
B1	>200	>200	>200	84	>200
B2	100	57.8∗	90	28∗	93.08
B3	>200	>200	>200	>200	>200
C1	104	53∗	NA	79∗	94.5
C2	125	101	78∗	78∗	91.6
C3	92	90	80	73∗	127
5FU	—	—	4.6∗∗	—	—
Tamoxifen	9.5∗∗	—	—	—	—
Betulinic acid	—	2.1∗∗	8.4∗∗	4.4∗∗	12.9∗∗

ND: not detected.

^a^The test for each extract was performed in triplicate and the results are presented as a mean percent inhibition ± SD. Each experiment was repeated three times (*n* = 3); ∗*P* < 0.05; ∗∗*P* < 0.01.

**Table 5:** GC-MS quantitative estimation of phytochemicals in SC-CO_2_ extract (B2) of *O. stamineus*.

Peak	Retention time (min)	Area %	Phytoconstituents	Molecular formula	Molecular weight
a	9.99	1.05	1,3-Benzodioxole, 5-[3-acetaminopropyl]-1-methyl-4-phenyl-3,4-dihydroisoquinoline myo-1,2,3,4,5,6-hexamethoxycarbonylcyclohexane	C_18_H_24_O_12_	432.375

b	11.15	1.34	2-Phenanthrenol, 1,2,3,4,4a,4b,5,6,8a,9,10,10a-dodecahydro-4a,7-dimethyl-8-[3-cyano-3(trimethylsilyloxy)propyl]-, acetate	C_25_H_41_NO_3_Si	431.69

c	12.19	1.24	2-Acetylamino-5-iodo-4-p-tolyl-thi ophene-3-carboxylic acid ethyl ester	C_18_H_54_O_9_Si_9_	667.385

d	13.13	1.66	Benzoic acid, 2,5-bis(trimethylsiloxy)-, trimethylsilyl ester	C_16_H_30_O_4_Si_3_	370.663

e	13.98	1.46	Acetamide, N-[(4.alpha.,5.alpha.)-cholestan-4-yl]	C_29_H_51_NO	429.721

f	14.78	2.35	Octadecamethyl-cyclononasiloxane	C_18_H_54_O_9_Si_9_	667.385

g	15.57	2.38	1-Monolinoleoylglycerol trimethylsilyl ether	C_27_H_54_O_4_Si_2_	498.886

h	16.48	6.65	2-Amino-2-oxo-acetic acid, N-[3,4-dimethylphenyl]-, ethyl ester	C_12_H_15_NO_3_	221.252

i	17.60	2.15	2-Propen-1-one, 1,3-diphenyl	C_15_H_12_O	208.26

J	25.33	27.84	Anthracene, 9-(2-propenyl)	C_17_H_14_	218.293

K	26.39	26.86	3,8-Dimethyl-4-(1-methylethylidene)-2,4,6,7,8,8a-hexahydro-5(1H)-azulenone	C_15_H_22_O	218.334

l	27.01	4.38	Acetic acid (3-hydroxy-7-isopropenyl-1,4a-dimethyl-2,3,4,4a,5,6,7,8-octahydronaphthalen-2-yl) ester	C_17_H_26_O_3_	278.386
